# Statin Use and Self-Reported Hindering Muscle Complaints in Older Persons: A Population Based Study

**DOI:** 10.1371/journal.pone.0166857

**Published:** 2016-12-02

**Authors:** Milly A. van der Ploeg, Rosalinde K. E. Poortvliet, Sophie C. E. van Blijswijk, Wendy P. J. den Elzen, Petra G. van Peet, Wouter de Ruijter, Jeanet W. Blom, Jacobijn Gussekloo

**Affiliations:** 1 Department of Public Health and Primary Care, Leiden University Medical Center, Leiden, the Netherlands; 2 Department of Clinical Chemistry and Laboratory Medicine, Leiden University Medical Center, Leiden, the Netherlands; University of Manitoba, CANADA

## Abstract

**Purpose:**

Statins are widely used by older persons in primary and secondary prevention of cardiovascular disease. Although serious adverse events are rare, many statin users report mild muscle pain and/or muscle weakness. It’s unclear what impact statins exert on a patient’s daily life. Research on statin related side effects in older persons is relatively scarce. We therefore investigated the relation between statin use and self-reported hindering muscle complaints in older persons in the general population.

**Methods:**

The present research was performed within the Integrated Systematic Care for Older Persons (ISCOPE) study in the Netherlands (Netherlands trial register, NTR1946). All registered adults aged ≥ 75 years from 59 participating practices (n = 12,066) were targeted. Information about the medical history and statin use at baseline and after 9 months was available for 4355 participants from the Electronic Patient Records of the general practitioners. In the screening questionnaire at baseline we asked participants: ‘At the moment, which health complaints limit you the most in your day-to-day life?’ Answers indicating muscle or musculoskeletal complaints were coded as such. No specific questions about muscle complaints were asked.

**Results:**

The participants had a median age of 80.3 (IQR 77.6–84.4) years, 60.8% were female and 28.5% had a history of CVD. At baseline 29% used a statin. At follow-up, no difference was found in the prevalence of self-reported hindering muscle complaints in statin users compared to non-statin users (3.3% vs. 2.5%, OR 1.39, 95% CI 0.94–2.05; P = 0.98). Discontinuation of statin use during follow-up was independent of self-reported hindering muscle complaints.

**Conclusion:**

Based on the present findings, prevalent statin use in this community-dwelling older population is not associated with self-reported hindering muscle complaints; however, the results might be different for incident users.

## Introduction

Statins, or 3-hydroxy-3-methylglutaryl coenzyme A (HMG CoA) reductase inhibitors, are among the most widely used drugs prescribed in the Western world [1]. Although serious adverse events (such as rhabdomyolysis) are relatively rare, statin use is associated with minor adverse events such as mild muscle pain and muscle weakness [2]. Depending on the definition of myopathy, the incidence of statin-related muscle symptoms is reported to range from 10–23% [2, 3]

Muscle symptoms are the main reason for discontinuation of statins and may exert more impact on a patient’s life than generally recognized [2, 4–7]. However, in randomized controlled trials (RCTs) mild muscle symptoms seemed to occur as frequently in patients on statins as on placebo [8,9].

Advancing age has been associated with increased risk of statin-induced muscle disorders. Also, the clinical effects of statin-associated muscle effects (e.g. functional impairment, risk of falls and/or disability) are likely to be greatest in older persons [10–11]. Most guidelines advice to start statin therapy for secondary cardiovascular prevention in older persons [12–14]. However, since most studies excluded patients aged ≥ 75 years [11] the risk-to-benefit ratio with advancing age becomes less clear [15].

Therefore, this study investigates the cross-sectional relationship between statin use and self-reported hindering muscle complaints in older persons in the general population.

## Methods

### Study design and participants

The present research was performed within the Integrated Systematic Care for Older Persons (ISCOPE) study in the Netherlands (Netherlands trial register, NTR1946)). The ISCOPE study is a pragmatic, cluster RCT comparing a proactive approach by the general practitioner (GP) with usual care provided by the GP, by monitoring the health status of older adults with complex problems. All registered adults aged ≥ 75 years from 59 participating practices (n = 12,066) were targeted. GPs excluded 590 persons who were deceased, too ill (i.e. terminally ill, acutely ill or patients with severe dementia), non-Dutch speaking, admitted to a nursing home, or otherwise judged unsuitable to participate.

The remaining 11,476 individuals received a written screening questionnaire, of which 7285 (63.5%) were completed and returned [16]. Due to organizational constraints, data on the medical history and statin use at baseline and after 9 months was obtained from electronic patient records (EPR) of a subgroup of the participating GPs. This resulted in data extraction for 4361 (59.9%) persons from the total 7285 participants; an additional 6 participants were excluded due to missing data, leaving 4355 participants eligible for the analyses. This subgroup is a representative sample of the total study population.

### Variables

#### Self-reported complaints causing hindrance in daily life

In the screening questionnaire at baseline we asked participants: ‘*At the moment*, *which health complaints limit you the most in your day-to-day life*?’ [17]. The answers were given in free text. We obtained our outcome variable ‘**muscle complaints’** by extracting all responses containing muscle complaints. For example, answers like ‘*My muscles are sore while walking*’ or ‘*Stiff muscles*. . .’ were coded as *‘***muscle complaints’**.

We used the outcome variable called *‘***musculoskeletal complaints’** for an additional analysis to check whether a broader definition of muscle complaints yielded different results. In addition to muscle complaints, this outcome also included complaints of pain in the neck, back, lower back, shoulder, upper extremities, lower extremities and/or joints. Examples of ‘**musculoskeletal complaints**’ reported by participants were ‘*My shoulder is painful*’ or ‘*My arms hurt’*. No specific questions about muscle complaints were asked.

#### Statin use

Patients using a statin at baseline were considered to be statin users. Those who weren’t were considered to be non-statin users. Information on statin use after 9 months follow-up was also available.

### Statistical analyses

We tested for differences between groups in categorical variables with Pearson’s chi-square test and Mann-Whitney-U test. Logistic regression techniques were used to test the association between statin use at baseline and self-reported hindering muscle complaints. We adjusted for age and sex. In the additional analyses we used logistic regression techniques to test the association between statin use at baseline and self-reported hindering musculoskeletal complaints. Rheumatoid arthritis, polymyalgia rheumatica or osteoarthritis are associated with muscle complaints, therefore, we excluded patients with these conditions for the outcomes ‘muscle complaints’ and ‘musculoskeletal complaints’ in a sensitivity analysis

The benefits of statin therapy as secondary prevention of cardiovascular disease (CVD) might be more pronounced than in primary prevention [18]. Therefore, we hypothesized that physicians might be less likely to continue statin therapy in the presence of hindering complaints in older patients without a history of CVD. This led us to perform a stratified analysis in participants with and without a history of CVD.

To investigate the relationship between self-reported hindering muscle complaints and discontinuation of statin therapy, we compared those who continued statin use for ≥ 9 months after baseline line and those who did use a statin at baseline but stopped within 9 months of baseline. We used Pearson’s chi-square test and logistic regression techniques controlling for age and sex.

The SPSS version 21.0 (SPSS Inc., Chicago, Ill., USA) was used to perform the statistical analyses.

### Ethical approval

The medical ethical committee of Leiden University Medical Center approved the Integrated Systematic Care for Older Persons (ISCOPE) study in 2009 including additional studies that could be performed with the collected data. Written informed consent was obtained from all participants.

## Results

### Basic characteristics

The median age of all participants was 80.3 (IQR 77.6–84.4) years, 60.8% were female and 28.5% had a history of CVD (s). At baseline, 1261 participants (29%) used a statin (Table 1). The most frequently used statin was simvastatin (53%), follow by atorvastatin (24%) and pravastatin (15%).

**Table 1:** Baseline Characteristics of The Study Population.

	All (n = 4355)	Statin use		
		No (n = 3094)	Yes (n = 1261)	p-value
Female (%)	2648 (60.8)	1974 (63.8)	674 (53.4)	< .001^a^
Age (median, IQR) years	80.3 (77.6–84.4)	80.6 (77.7–85.0)	79.8 (77.2–83.1)	< .001^b^
History of cardiovascular disease (%)	1243 (28.5)	556 (18.0)	687 (55.3)	< .001^a^

IQR = interquartile range

^a^p-value is calculated using Pearson’s chi-square test

^b^p-value is calculated using Mann-Whitney U test

Non-statin users were slightly older than statin users (median age 80.6 vs 79.8 years; P < .001). Males were more likely to use statins than females (P < .001). A history of CVD was associated with statin use (P < .001) (see Table 1).

#### Hindering muscle complaints

**Fig 1:**
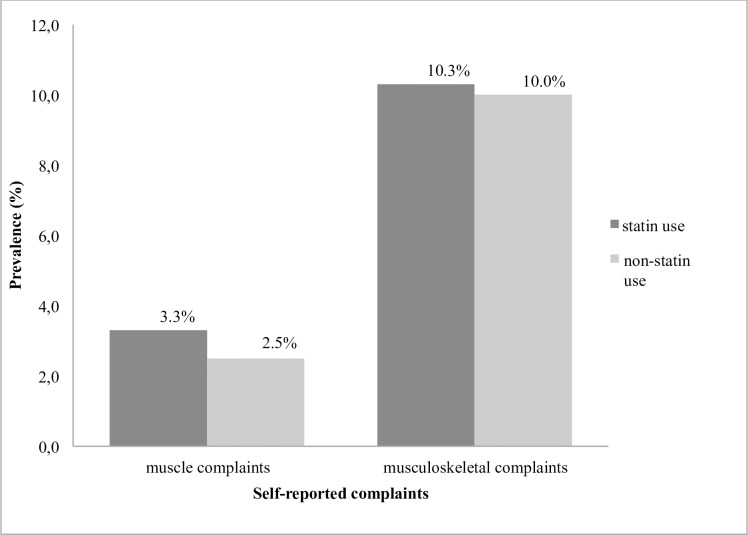
Prevalence of self-reported complaints according to statin use and non-statin use.

Hindering muscle complaints were reported by 2.7% of the participants. There was no difference in the prevalence of hindering muscle complaints between statin users and non-statin users (3.3% vs 2.5%, P = 0.18) (see Fig 1). After adjustment for age and sex there was no difference in the prevalence of muscle complaints between the two groups (OR_statin use_ = 1.39, 95% CI 0.94–2.05; P = 0.98). Similar results emerged when participants with a history of rheumatoid arthritis, polymyalgia rheumatic and osteoarthrosis (n = 1115) were excluded (OR_statin use_ = 1.28, 95% CI 0.80–2.07; P = 0.30). An additional analysis for musculoskeletal complaints yielded similar results (Fig 1).

### History of cardiovascular disease

There was no difference in self-reported complaints between participants using statins and participants not using statins, stratified by history of CVD. In both strata the prevalence of self-reported muscle complaints was independent of statin use (OR_statin use_ = 1.20, 95% CI 0.68–2.10; P = 0.53 for no history of CVD, and OR_statin use_ = 1.31, 95% CI 0.68–2.53; P = 0.42 for history of CVD). Absolute differences in prevalence between statin users and non-statin users were 0.4% (2.8% in statin-users and 2.4% in non-statin users) in participants without a history of CVD, and 0.7% (3.6% in statin users and 2.9% in non-statin users) in those with a history of CVD.

### Discontinuation of statin use

Of the 1261 participants using a statin at baseline, 118 (9.4%) discontinued statin use within 9 months. There was no difference in the prevalence of muscle complaints among those who discontinued statins compared to continuous users (5.1% and 3.1%, respectively; P = 0.18). After adjustment for age and sex, the prevalence of self-reported muscle complaints showed no significant difference between participants who continued or stopped using a statin (OR = 1.70, 95% CI 0.70–4.13; P = 0.24). Additional analysis for musculoskeletal complaints yielded similar results (data not shown).

## Discussion

This observational study investigated whether statin use in old age is associated with self-reported hindering muscular complaints in daily life. We found no significant difference between the prevalence of self-reported hindering muscle complaints in statin users compared to non-statin users. Also, we found that discontinuation of statin use within 9 months after baseline was independent of self-reported hindering muscle complaints; moreover, there was no evidence that hindering muscular complaints were a reason for discontinuing statin therapy.

### Muscle complaints

Our results are in line with most RCTs on statins, showing that statin use is not related to an increased risk of musculoskeletal symptoms [8]. In contrast, observational studies have reported a relationship between statin use and muscular symptoms interfering with daily activities. [2,19,20]^.^ Explanations for the different findings in RCTs versus observational studies include various sources of selection bias (e.g. sampling bias) [8].

One explanation for the negative results of the present study could be that persons with hindering complaints might have discontinued statin therapy before the data were extracted from the EPR at baseline. It is known that the time of onset of muscular side-effects is relatively short (usually within months after initiation of treatment or titration to a high dosage) and that statin-associated muscle symptoms are the main reason for discontinuation of statin treatment [2,4,6,7,21]. This might have resulted in underestimation of the association between statin use and muscle complaints in the present study. This type of bias (under ascertainment of events/side-effects that occur early in therapy) is a well-known weakness of observational studies and often occurs when including prevalent users [22]. By comparing the self-reported complaints in participants who discontinued statin treatment within 9 months after baseline with participants who continued treatment, we aimed to account for this bias. Because no difference was found between the groups, this makes underestimation of the true effect less likely to be substantial.

Another explanation for our findings is that statins are not (or not as strongly) related with muscle symptoms as generally thought. The debate on statin-related muscle symptoms originates from the withdrawal of cerivastatin from the market because of safety concerns and its association with rhabdomyolysis [23]. The debate continues regarding the frequency and severity of statin-related side-effects [24,25].

The media has paid considerable attention to statin-related muscle symptoms, which may have led to a phenomenon referred to as the ‘nocebo effect’. This implies that when patients have negative expectations about a drug they can experience unpleasant effects that they attribute to the drug [26,27]. From this viewpoint, some of the reported muscle symptoms of patients might be explained by the expected association between statins and the occurrence of muscle symptoms. Also, physicians might be inclined to, erroneously, attribute muscle symptoms to statin use [8]; in fact, our results show that hindering muscle complaints are relatively common among older persons, irrespective of the use of statins.

Finally, our study focused on self-reported *hindering* complaints. Therefore, statin users could have experienced more muscle symptoms than actually reported during this study, but did not perceive them as being hindering in their daily life. In this case, statin-related muscle complaints might not exert such a large impact on the daily life of older persons, as also reported by others [2,28].

### History of cardiovascular disease

Our hypothesis that physicians might be less likely to continue statin therapy in the presence of hindering complaints in older patients without a history of CVD compared to patients with a history of CVD could not be confirmed. After stratification for a history of CVD the absence of an association between statin use and the prevalence of muscle complaints was shown to be independent of a history of CVD. This could be due to the absence of a true effect, or to the decrease in group size and, therefore, loss of statistical power.

### Strengths and limitations

This study has several strengths. First, the participants were community-dwelling older persons providing a representative sample of older statin users in daily practice.

Second, the mean age of our study population was relatively high. Although studies in this age group are relatively scarce, older persons frequently use statins and advancing age is associated with a higher risk of statin-induced muscle disorders [29]. Therefore, this study provides valuable information on the risks and benefits of frequently prescribed drugs, such as statins, in older persons.

As mentioned above, the most important limitation of this study is that our results might underestimate the true effect due to the prevalent-user design; this could have led to a type II error.

In The Netherlands 1.200.000 persons are 75 years and older [30]. In total 248.730 persons use lipid lowering drugs [31]. The prevalence of statin-use in our study (29%) is therefore slightly higher compared to the use in the general population (21%). Since there is more evidence of a favorable risk-to-benefit ratio in younger individuals it is likely that statins are more often prescribed to people under 75 years than people over 75 years of age. The slightly higher prevalence of statin-use in our population could be related to the exclusion of patients groups that might be less likely to use a statins because of questionable benefits (participants that are terminally ill or with severe dementia). Demographic variation and chance could also play a role.

Most of our participant (92%) on statin therapy used either simvastatin, atorvastatin of pravastatin. This distribution is likely to represent that of the general population in The Netherlands since simvastatin and atorvastatin are the preferred statins according to the widely used national guidelines [32]. However in other countries this might be different.

Because of our in- and exclusion criteria we cannot extrapolate our conclusions to younger age groups nor to patients who are terminally ill or patients with severe dementia.

In addition to statin-use other factors and conditions (i.e. like congenital disorders or sustained trauma) could cause musculoskeletal complaints. However, we assume that these conditions are not more (or less) prevalent in participants using statins compared to participants not using statins making them unlikely confounding factors. Also, when we excluded participants with a history of rheumatoid arthritis, polymyalgia rheumatic and osteoarthrosis from the analysis, again we found that statin-use was not related to muscle or musculoskeletal complaints.

Furthermore, inherent to our cross-sectional study design, no conclusions about causality can be drawn. With the current data we were unable to investigate the influence of the duration of statin use, dose-related effects, or differences between types of statins. Since statin-related muscle symptoms have been reported in patients on high-dose statin therapy [21] this is a worthwhile topic for further research.

## Conclusion

Based on the present findings, prevalent statin use in this community-dwelling older population is not associated with self-reported hindering muscle complaints; however, the results might be different for incident users.

## References

[1] IMS Institute for Healthcare Informatics. The use of medicines in the United States: review of 2011. Available from: https://www.imshealth.com/files/web/IMSH%20Institute/Reports/The%20Use%20of%20Medicines%20in%20the%20United%20States%202010/Use_of_Meds_in_the_U.S._Review_of_2010.pdf

[2] Rosenbaum D, Dallongeville J, Sabouret P, Bruckert E. Discontinuation of statin therapy due to muscular side effects: a survey in real life. Nutr Metab Cardiovasc Dis 2013;23(9):871–875. 10.1016/j.numecd.2012.04.012 22748604

[3] Joy TR, Hegele RA. Narrative review: statin-related myopathy. Ann Intern Med 2009;150(12):858–868. 19528564 10.7326/0003-4819-150-12-200906160-00009

[4] Chodick G, Shalev V, Gerber Y, Heymann AD, Silber H, Simah V, et al Long-term persistence with statin treatment in a not-for-profit health maintenance organization: a population-based retrospective cohort study in Israel. Clin Ther 2008;30(11):2167–2179. 10.1016/j.clinthera.2008.11.012 19108805

[5] Chowdhury R, Khan H, Heydon E, Shroufi A, Fahimi S, Moore C, et al Adherence to cardiovascular therapy: a meta-analysis of prevalence and clinical consequences. Eur Heart J 2013;34(38):2940–2948. 10.1093/eurheartj/eht295 23907142

[6] Stroes ES, Thompson PD, Corsini A, Vladutiu GD, Raal FJ, Ray KK, et al Statin-associated muscle symptoms: impact on statin therapy-European Atherosclerosis Society Consensus Panel Statement on Assessment, Aetiology and Management. Eur Heart J 2015;36(17):1012–1022. 10.1093/eurheartj/ehv043 25694464 PMC4416140

[7] Cohen JD, Brinton EA, Ito MK, Jacobson TA. Understanding Statin Use in America and Gaps in Patient Education (USAGE): an internet-based survey of 10,138 current and former statin users. J Clin Lipidol 2012;6(3):208–215. 10.1016/j.jacl.2012.03.003 22658145

[8] Finegold JA, Manisty CH, Goldacre B, Barron AJ, Francis DP. What proportion of symptomatic side effects in patients taking statins are genuinely caused by the drug? Systematic review of randomized placebo-controlled trials to aid individual patient choice. Eur J Prev Cardiol 2014;21(4):464–474. 10.1177/2047487314525531 24623264

[9] Law M, Rudnicka AR. Statin safety: a systematic review. Am J Cardiol 2006 17;97(8A):52C–60C. 10.1016/j.amjcard.2005.12.010 16581329

[10] Bhardwaj S, Selvarajah S, Schneider EB. Muscular effects of statins in the elderly female: a review. Clin Interv Aging 2013;8:47–59. 10.2147/CIA.S29686 23355775 PMC3552608

[11] Rich MW. Aggressive lipid management in very elderly adults: less is more. JAGS 2014;62(5):945–947. 10.1111/jgs.12788_2 24801130

[12] Stone NJ, Robinson JG, Lichtenstein AH, Bairey Merz CN, Blum CB, Eckel RH, et al 2013 ACC/AHA guideline on the treatment of blood cholesterol to reduce atherosclerotic cardiovascular risk in adults: a report of the American College of Cardiology/American Heart Association Task Force on Practice Guidelines. J Am Coll Cardiol 2014;63(25 Pt B):2889–2934. 24239923 10.1016/j.jacc.2013.11.002

[13] Perk J, De Backer G, Gohlke H, Graham I, Reiner Z, Verschuren M, et al European Guidelines on cardiovascular disease prevention in clinical practice (version 2012). The Fifth Joint Task Force of the European Society of Cardiology and Other Societies on Cardiovascular Disease Prevention in Clinical Practice (constituted by representatives of nine societies and by invited experts). Eur Heart J 2012;33(13):1635–1701. 10.1093/eurheartj/ehs092 22555213

[14] National Clinical Guideline Centre (UK). Lipid Modification: Cardiovascular Risk Assessment and the Modification of Blood Lipids for the Primary and Secondary Prevention of Cardiovascular Disease. London: National Institute for Health and Care Excellence (UK); 2014 7 (NICE Clinical Guidelines, No. 181) Available from: http://www.ncbi.nlm.nih.gov/books/NBK248067/25340243

[15] Petersen LK, Christensen K, Kragstrup J. Lipid-lowering treatment to the end? A review of observational studies and RCTs on cholesterol and mortality in 80+-year olds. Age Ageing 2010;39(6):674–680. 10.1093/ageing/afq129 20952373 PMC2956535

[16] Blom JW, den Elzen WPJ, Van Houwelingen AH, Heijmans M, Stijnen T, Van den Hout W, et al Effectiveness and cost-effectiveness of a proactive, goal-oriented, integrated care model in general practice for older people. A cluster randomized controlled trial: Integrated Systematic Care for older People–the ISCOPE study. Age Ageing 2016;45(1):30–41. 10.1093/ageing/afv174 26764392 PMC4711660

[17] van Blijswijk SC, Chan OY, van Houwelingen AH, Gussekloo J, den Elzen WP, Blom JW. Self-Reported Hindering Health Complaints of Community-Dwelling Older Persons: A Cross-Sectional Study. PloS One 2015;10(11):e0142416 10.1371/journal.pone.0142416 26571233 PMC4646486

[18] Shepherd J, Blauw GJ, Murphy MB, Bollen EL, Buckley BM, Cobbe SM, et al Pravastatin in elderly individuals at risk of vascular disease (PROSPER): a randomised controlled trial. Lancet 2002;360(9346):1623–1630. 12457784 10.1016/s0140-6736(02)11600-x

[19] Thompson PD, Parker BA, Clarkson PM, Pescatello LS, White CM, Grimaldi AS, et al A randomized clinical trial to assess the effect of statins on skeletal muscle function and performance: rationale and study design. Prev Cardiol 2010;13(3):104–111. 10.1111/j.1751-7141.2009.00063.x 20626664 PMC4107659

[20] Parker BA, Capizzi JA, Grimaldi AS, Clarkson PM, Cole SM, Keadle J, et al Effect of statins on skeletal muscle function. Circulation 2013;127(1):96–103. 10.1161/CIRCULATIONAHA.112.136101 23183941 PMC4450764

[21] Bruckert E, Hayem G, Dejager S, Yau C, Begaud B. Mild to moderate muscular symptoms with high-dosage statin therapy in hyperlipidemic patients—the PRIMO study. Cardiovasc Drugs Ther 2005;19(6):403–414. 10.1007/s10557-005-5686-z 16453090

[22] Ray WA. Evaluating medication effects outside of clinical trials: new-user designs. Am J Epidemiol 2003;158(9):915–920. 14585769 10.1093/aje/kwg231

[23] Omar MA, Wilson JP, Cox TS. Rhabdomyolysis and HMG-CoA reductase inhibitors. Ann Pharmacother 2001;35(9):1096–1107. 11573861 10.1345/aph.10228

[24] Zhang H, Plutzky J, Skentzos S, Morrison F, Mar P, Shubina M, et al Discontinuation of statins in routine care settings: a cohort study. Ann Intern Med 2013;158(7):526–534. 10.7326/0003-4819-158-7-201304020-00004 23546564 PMC3692286

[25] Abramson JD, Rosenberg HG, Jewell N, Wright JM. Should people at low risk of cardiovascular disease take a statin? BMJ 2013;347:f6123 10.1136/bmj.f6123 24149819

[26] Barsky AJ, Saintfort R, Rogers MP, Borus JF. Nonspecific medication side effects and the nocebo phenomenon. JAMA. 2002;287(5):622–627. 11829702 10.1001/jama.287.5.622

[27] Colloca L, Finniss D. Nocebo effects, patient-clinician communication, and therapeutic outcomes. JAMA 2012;307(6):567–568. 10.1001/jama.2012.115 22318275 PMC6909539

[28] Cham S, Evans MA, Denenberg JO, Golomb BA. Statin-associated muscle-related adverse effects: a case series of 354 patients. Pharmacotherapy 2010;30(6):541–553. 10.1592/phco.30.6.541 20500044 PMC4729295

[29] Alexander KP, Blazing MA, Rosenson RS, Hazard E, Aronow WS, Smith SC Jr, et al Management of hyperlipidemia in older adults. J Cardiovasc Pharmacol Ther 2009;14(1):49–58 10.1177/1074248408328927 19124599

[30] http://www.eengezondernederland.nl/Trends_in_de_toekomst/Determinanten (accessed 01-08-2016).

[31] https://www.gipdatabank.nl/databank.asp?tabel=03-lftgesl&geg=gebr&item=C10 (accessed 01-08-2016)

[32] Cardiovasculair risicomanagement (Tweede herziening) Huisarts Wet 2012;55(1):14–28. Available from: https://www.nhg.org/standaarden/volledig/cardiovasculair-risicomanagement#Begrippen

