# Correction: Statin Use and Self-Reported Hindering Muscle Complaints in Older Persons: A Population Based Study

**DOI:** 10.1371/journal.pone.0245997

**Published:** 2021-01-22

**Authors:** Milly A. van der Ploeg, Rosalinde K. E. Poortvliet, Sophie C. E. van Blijswijk, Wendy P. J. den Elzen, Petra G. van Peet, Wouter de Ruijter, Jeanet W. Blom, Jacobijn Gussekloo

In the Results subsection of the Abstract, there is an error in the P-value in the second sentence. The correct sentence is: At follow-up, no difference was found in the prevalence of self-reported hindering muscle complaints in statin users compared to non-statin users (3.3% vs. 2.5%, OR 1.39, 95% CI 0.94–2.05; P = 0.10).

In the Hindering muscle complaints subsection of the Results, there is an error in the P-value in the third sentence. The correct sentence is: After adjustment for age and sex there was no difference in the prevalence of muscle complaints between the two groups (OR_statin use_ = 1.39, 95% CI 0.94–2.05; P = 0.10).
